# Magnetoencephalographic study of event‐related fields and cortical oscillatory changes during cutaneous warmth processing

**DOI:** 10.1002/hbm.23977

**Published:** 2018-01-23

**Authors:** Kyung‐min An, Sanghyun Lim, Hyun Joon Lee, Hyukchan Kwon, Min‐Young Kim, Bakul Gohel, Ji‐Eun Kim, Kiwoong Kim

**Affiliations:** ^1^ Center for Biosignals, Korea Research Institute of Standards and Science (KRISS) Daejeon Republic of Korea; ^2^ Department of Medical Physics University of Science and Technology (UST) Daejeon Republic of Korea; ^3^ Department of Physics Pusan National University Busan Republic of Korea

**Keywords:** alpha, beta, cortical oscillations, delta, event‐related fields, laser stimulation, magnetoencephalography, warmth

## Abstract

Thermoreception is an important cutaneous sense, which plays a role in the maintenance of our body temperature and in the detection of potential noxious heat stimulation. In this study, we investigated event‐related fields (ERFs) and neural oscillatory activities, which were modulated by warmth stimulation. We developed a warmth stimulator that could elicit a warmth sensation, without pain or tactile sensation, by using a deep‐penetrating 980‐nm diode laser. The index finger of each participant (*n* = 24) was irradiated with the laser warmth stimulus, and the cortical responses were measured using magnetoencephalography (MEG). The ERFs and oscillatory responses had late latencies (∼1.3 s and 1.0–1.5 s for ERFs and oscillatory responses, respectively), which could be explained by a slow conduction velocity of warmth‐specific C‐fibers. Cortical sources of warmth‐related ERFs were seen in the bilateral primary and secondary somatosensory cortices (SI and SII), posterior part of the anterior cingulate cortex (pACC), ipsilateral primary motor, and premotor cortex. Thus, we suggested that SI, SII, and pACC play a role in processing the warmth sensation. Time–frequency analysis demonstrated the suppression of the alpha (8–13 Hz) and beta (18–23 Hz) band power in the bilateral sensorimotor cortex. We proposed that the suppressions in alpha and beta band power are involved in the automatic response to the input of warmth stimulation and sensorimotor interactions. The delta band power (1–4 Hz) increased in the frontal, temporal, and cingulate cortices. The power changes in delta band might be related with the attentional processes during the warmth stimulation.

## 1 INTRODUCTION

The skin protects our body, and as a sensory organ, it plays a role in detecting mechanical, chemical, and thermal stimuli from the environment. The sense of warmth is important to obtain thermal information for homeostasis and to detect potentially noxious heat stimulation. Whereas other cutaneous sensations are being actively investigated, the study of innocuous warmth sensation has been limited, due to the weak cortical responses to warmth stimulation and the limitations of the warmth stimulator (Chang, Arendt‐Nielsen, & Chen, 2005).

Innocuous warmth activates thermal receptors called transient receptor potential vanilloid‐3 (TRPV‐3) ion channels (34–38°C) and TRPV‐4 ion channels (27–35°C) in the dermis and epidermis (Gûler et al., 2002; Patapoutian, Peier, Story, & Viswanath, 2003; Pogorzala, Mishra, & Hoon, 2013; Tominaga & Calerina, 2004). Input from warmth stimuli is transmitted through warmth‐specific C‐fibers (Darian‐Smith et al., 1979; Iggo, 1984). As warmth‐specific C‐fibers have an unmyelinated thin axon (0.2–1.5 μm), they have slow conduction velocities of ∼0.5–2 m/s (Siegel & Sapru, 2006). It has been well known that warmth stimulation generates event‐related potentials (ERPs) with late latencies (Cruccu et al., 2003; Granovsky, Matre, Sokolik, Lorenz, & Casey, 2005; Iannetti et al., 2003; Valeriani et al., 2011, 2002).

C‐fibers, together with Aδ‐fibers, are termed as *small fibers*. C‐fibers deliver warmth sensation and dull pain, while Aδ‐fibers deliver acute pain. These small fibers can be damaged in patients who are old or have a metabolic disorder. The neural disorder caused by the degeneration of the small fibers is called small fiber neuropathy (SFN). Painful stimuli are usually used to examine functions of Aδ‐fibers to reach a diagnosis of SFN, but the criteria for the diagnosis of SFN is not yet well established (Devigili et al., 2008; Terkelsen et al., 2017). Compared with Aδ‐fibers, C‐fibers have small receptive fields and sparse innervations. Owing to the low density of the C‐fibers, the dysfunctions of C‐fibers can directly lead to the loss of warmth sensing ability in the local skin area, while the dysfunctions of Aδ‐fibers can be compensated by the neighboring Aδ‐fibers. Thus, measuring the dysfunction of C‐fibers, rather than Aδ‐fibers, might be more effective for an early diagnosis of SFN (Schmelz, 2011).

The exact brain areas that are related to the information processing of the warmth stimulation delivered through C‐fibers currently remain controversial. Some of the previous electroencephalography (EEG) studies reported that the posterior part of the anterior cingulate cortex (pACC) and secondary somatosensory cortex (SII), but not the primary somatosensory cortex (SI), play a role in warmth sensation (Cruccu et al., 2003; Iannetti et al., 2003). Other functional magnetic resonance imaging (fMRI) studies, however, demonstrated the activation of both SI and SII during warmth stimulation (Moulton, Keaser, Gullapalli, & Greenspan, 2005; Peltz et al., 2011). As both SI and SII are involved in processing of other cutaneous senses, further multimodal study is required to explain the discrepancy between fMRI and EEG studies.

In recent studies, the oscillatory responses of the brain are considered to be related to cognitive operations, perceptions, and sensorimotor functions (Başar, Başar‐Eroğlu, Karakaş, & Schürmann, 2000; Klimesch, 1999; Palva & Palva, 2007). While the oscillatory activities in various frequency bands during tactile (Michail, Dresel, Witkovský, Stankewitz, & Schulz, 2016) or pain stimulation (Dowman, Rissacher, & Schuckers, 2008; Gross, Schnitzler, Timmermann, & Ploner, 2007; Iannetti, Hughes, Lee, & Mouraux, 2008; Michail et al., 2016; Nir, Sinai, Moont, Harari, & Yarnitsky, 2012; Ploner, Gross, Timmermann, Pollok, & Schnitzler, 2006; Raij, Forss, Stancák, & Hari, 2004) have been actively investigated, frequency‐specific neural oscillatory activities related to the warmth stimuli are not well understood yet. Stančák, Poláček, Vrána, and Mlynář (2007) analyzed oscillatory power changes during ramp warming, at 10 and 20 Hz, using EEG. When the warmth stimulus was applied, 10 and 20 Hz oscillations in the contralateral sensorimotor cortex and premotor cortex decreased. The amplitude of 20 Hz oscillations, however, increased in the anterior cingulate cortex (ACC) and ipsilateral premotor cortex.

In this study, we investigated cortical responses to warmth stimulation by using MEG. In comparison to fMRI and positron emission tomography, MEG and EEG can record neural activity at a higher temporal resolution. MEG has a higher spatial resolution compared with EEG (Srinivasan, Winter, Ding, & Nunez, 2007), and less distorted by the brain tissues (e.g., skin, skull, white matter, gray matter, and cerebrospinal fluid). To elicit pure warmth sensation, without pain or tactile sense, we developed a warmth stimulator using a 980 nm diode laser stimulator.

The purpose of this study was to investigate the phase‐locked (ERFs) and phase‐unlocked (oscillatory power changes) cortical activity related to warmth stimulation. We analyzed the topographical and cortical source mapping of the ERFs and oscillatory power changes in various frequency bands during the warmth information processing.

## 2 MATERIALS AND METHODS

### 2.1 Participants

MEG data were obtained from 30 healthy right‐handed volunteers with normal or corrected to normal vision (15 women and 15 men; 20 years to 27 years [22.6 ± 2.2] [mean ± SD]). All participants gave their written informed consent. The experimental procedures were approved by the ethics committee of the Korea Research Institute of Standard and Science (KRISS‐IRB 2015‐3). None of the participants suffered from diseases, which could affect normal somesthetic perception.

### 2.2 Warmth stimulator

A diode laser tuned to 980 nm was used as a warmth stimulator. As the laser increased the temperature of the skin fast (Arendt‐Nielsen & Chen, 2003), the evoked brain response could be obtained with short duration laser pulse stimuli. In the preliminary experiment, we had measured the skin temperature using an IR thermometer (CT09, Infrared radiation pyrometer, HEITRONICS, Germany). We found that the 980‐nm laser rapidly increased the skin temperature during the laser exposure time (Supporting Information, Figure S1). The laser increased the temperature of the skin without physical contact (Gülsoy, Durak, Kurt, Karamürsel, & Çilesiz, 2001), and the warmth stimulation was delivered without tactile cutaneous stimulation. CO_2_ laser has been popularly used as laser stimulations to elicit heat pain. Some studies, however, reported that a CO_2_ laser could cause transiently lasting thermal damage at high energies, as the CO_2_ laser beam can penetrate the superficial layer of skin (Arendt‐Nielsen & Chen, 2003; Towell, Purves, & Boyd, 1996). In this study, we used a diode laser tuned to 980 nm, which enables much longer skin penetration depth without inducing skin damage due to the lower absorption in tissue than the CO_2_ laser.

**Figure 1:**
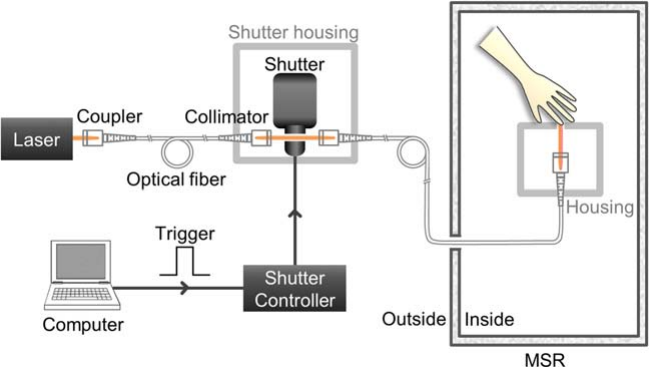
Experimental schematic of the laser‐based warmth stimulator. A diode laser tuned to 980 nm and shutter system were placed outside a magnetically shielded room (MSR). The stimulus presenting part alone was placed inside the room to minimize the effects of external magnetic noise. A laser beam was delivered to the index finger of the participant, who was seated in the MSR. A computer was used to trigger the shutter controller. The mechanical shutter and shutter controller were used to adjust the stimulus duration. For the safety of the participants, the mechanical shutter and stimulus presenting part were shielded with aluminum and polytetrafluoroethylene, respectively [Color figure can be viewed at http://wileyonlinelibrary.com]

Figure 1 shows the schematic diagram of the warmth stimulator that was developed for our experiment. The laser beam was delivered to the ventral side of the left index finger of participants in a magnetically shielded room (MSR) through optical fibers. Most parts of the stimulator, except for the stimulus presenting part, were located outside of the MSR through optical fibers, which were 2.5 m in length, to minimize the magnetic noise from the stimulator. The duration of the laser beam irradiation was controlled with a shutter system using a trigger input from a remote control. The mechanical shutter and stimulus presenting part were shielded with aluminum and polytetrafluoroethylene, respectively, for the safety of the participants.

### 2.3 Experimental paradigm

**Figure 2:**
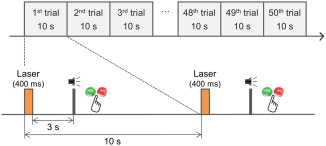
Experimental paradigm for one recording session in the warmth study. We conducted three successive sessions, and each session consisted of 50 trials. In each trial, the pulsed diode laser beam was applied to the left index finger, for a duration of 400 ms, with a 10‐s inter‐stimulus interval. We delivered a 500‐Hz auditory beep sound 3 s after each laser stimulation and asked the participants to evaluate if the warmth stimulation was perceived or not, by pressing either “yes” or “no” button, respectively, after each auditory beep sound [Color figure can be viewed at http://wileyonlinelibrary.com]

Figure 2 shows the experimental paradigm for one session for the current study. During each session, the warmth stimulation was delivered to the participant's left index finger 50 times, with an interstimulus interval of 10 s to minimize habituation and skin irritation. Participants were instructed to press either “yes” or “no” button by their right index finger after the auditory beep sound to evaluate whether they could perceive the warmth sensation or not. A 500‐Hz auditory beep sound was delivered 3 s after each warmth stimulus as a cue to evaluate warmth perception, while it minimizes the temporal overlap between the warmth sensation and finger movement execution. We conducted three successive sessions for MEG recording on the same day. The interval between the sessions was about 3 min. When the participants insisted on continuing the measurement, we recorded the next session without a break. Previous researches used laser stimuli with a longer duration and larger beam area to elicit the warmth sensation, compared with those used to elicit a pain sensation (Agostino et al., 2000; Cruccu et al., 2003; Iannetti et al., 2003; Valeriani et al., 2002). In this study, we used a laser pulse with a 400‐ms duration and a cross‐sectional beam diameter of 6 mm. We assessed individual warmth sensitivity using a warmth threshold estimation procedure included before each recording session. During the warmth threshold estimation procedure, laser pulses were irradiated 30 times with a 10‐s interstimulus interval, and participants were asked to evaluate each laser stimulation. We determined the laser intensity at which the participant perceived pure warmth sensations, ∼80% more than the warmth threshold. The individually averaged laser intensity, which was applied to elicit warmth sensations without the perception of pain, is summarized in the Supporting Information, Table S1. Furthermore, the Supporting Information, Table S1 outlined the individually averaged warmth perception rate, at which they felt a sensation of pure warmth without pain during the experimental session. The averaged laser intensity for the warmth stimuli across all participants was 59.4 ± 15.5 (mean ± SD) mJ/mm^2^. Gülsoy et al. (2001) reported that the participants felt a pinprick of pain when the intensity of the laser irradiation was approximately 212.7 mJ/mm^2^, with a 980‐nm wavelength. The laser used in this previous study, however, was ∼3.5 times larger than that used for warmth stimuli in the current experiment.

### 2.4 Magnetoencephalography (MEG) recordings

The brain responses to the warmth sensation were measured using an MEG system (152 channels axial gradiometer), which was developed by the Korea Research Institute of Standards and Science in South Korea (Kim et al., 2014; Lee et al., 2009). The magnetoencephalographic signals were recorded using a DC‐234 Hz pass filter, and digitized at a sampling rate of 1024 Hz. All experiments were conducted in an MSR. The room temperature and humidity were maintained at 21°C–23°C and 63%–67%, respectively. Participants were seated in a comfortable armchair and were instructed to keep their eyes open and gaze at the fixation point displayed on the screen located ∼80 cm away.

Before the experiment, participants received a detailed explanation of the procedure and were familiarized with the experimental surrounding and test stimuli. Four head positioning coils were mounted to estimate the head locations of the participant in the MEG helmet. Locations of positioning coils were recorded before and after each session. The anatomical fiducial points (i.e., nasion, and left and right preauricular), the center points of each head positioning coil, and 45 head surface points were measured using a 3D digitizer (ISOTRAK, Polhemus Navigation, Colchester, USA). Participants then underwent the MEG experiment consisting of three successive sessions with 50 trials in each session (Figure 2).

### 2.5 Data analysis

For analysis of the MEG data, we used Fieldtrip for data preprocessing and time‐frequency analysis (Oostenveld, Fries, Maris, & Schoffelen, 2011; http://www.ru.nl/fcdonders/fieldtrip/) and Brainstorm for source analysis (Tadel, Baillet, Mosher, Pantazis, & Leahy, 2011). Open source toolboxes were run using a Matlab (The Mathworks, Natick, Massachusetts, USA) environment. Continuous MEG data were band‐pass filtered between 0.02 and 100 Hz and segmented from −3 to 5 s with respect to the stimulus onset. To reject artifacts due to eye blinks, eye movements, and heart beats, we applied an independent component analysis method (“runica” implemented in FieldTrip, http://www.sccn.ucsd.edu/eeglab/). Independent components representing the ocular and cardiac activities were identified by visual inspection based on their topography and their time‐course. The artifact‐rejected waveforms were recovered by back‐projection of the rest independent components into the signal‐space after eliminating these artifact components. We selected trials where the participants pressed the “yes” button and applied the 40‐Hz low‐pass filter. The trials with muscle artifacts were rejected. Further, we discarded data from 6 participants who had <120 trials. Data from 24 participants in total (13 women and 11 men) were analyzed. The relative head positions of the MEG sensors were different across sessions. The MEG data were interpolated to standard sensor locations, which was obtained by averaging the sensor positions over all the sessions across subjects by using the “ft_megrealign” function from the Fieldtrip.

To obtain ERFs evoked by the warmth stimulation, we averaged all the trials from the three successive sessions, preprocessed artifact‐free trials for each participant, and examined the grand‐average for the entire participants. The baseline was selected from −1.1 to −0.1 s prior to the stimulus onset. To obtain the source activity of warmth‐related ERFs in the brain region, we performed the weighted minimum norm estimates (wMNE) (Hamalainen & Ilmoniemi, 1994; Hauk, 2004; Lin et al., 2006), which is implemented in Brainstorm toolbox (Tadel et al., 2011). We used the ICBM152 template anatomy, which was scaled according to each participant's individual head shapes, in the Brainstorm toolbox. The baseline period (−1.1 to −0.1 s) was used to estimate noise‐covariance for each session of each subject. The wMNE source analysis was performed on an overlapping‐sphere head model with standard Tikhonov regularization (λ = 0.1).

Before the time–frequency analysis, we applied the planar transformation for easier interpretation of MEG signals with complicated spatial patterns. Preprocessed data based on axial gradiometer was transformed to planar gradient sensors, using “ft_megplanar” and “ft_combineplanar” functions from the fieldtrip toolbox, to locate oscillatory brain activation more easily. We calculated time–frequency representations (TFRs) at 2–40 Hz, using a sliding Hanning‐window Fourier transform approach with a fixed 500‐ms time window, moving in steps of 10 ms. The results of TFRs were expressed as percent power change relative to baseline (i.e., −1.1 to −0.1 s). The TFRs of each sensor were grand‐averaged across participants.

For cortical mapping of oscillatory power changes, we filtered the wMNE source data in specific frequency ranges (i.e., 1–4 Hz, 4–8 Hz, 8–13 Hz, and 18–23 Hz, for delta, theta, alpha, and beta, respectively). Thereafter, we applied the Hilbert transformation to obtain power and phase information in the cortical sources.

### 2.6 Statistical analysis

Next, we assessed the significance of cortical activations of the ERFs related to the warmth stimulation by comparing the activated (1.2–1.4 s) and baseline (−0.3 to −0.1 s) periods using a paired *t* test, with Bonferroni multiple corrections.

Time–frequency windows of interest for each frequency component were selected at the maximum power of the oscillatory responses as follows: delta activity at 1–4 Hz (between 1.0 and 1.5 s), alpha activity at 8–13 Hz (between 1.0 and 1.5 s), and beta activity at 18–23 Hz (between 1.1 and 1.3 s). We used a cluster‐based permutation test for the distributions of ERFs and oscillatory band powers in the sensor space to assess differences between the activated period and baseline period. We used a paired *t* test to compare cortical source activations of the oscillatory band powers between the activated and baseline periods; the false discovery rate (FDR) correction was applied to control for the type I error.

## 3 RESULTS

### 3.1 Event‐related fields elicited by warmth stimulation

All participants reported feeling the stimulation as a “slightly warm” or “warm” sensation. There were no reports of a “burning” or “pinprick” pain sensation. Participants showed a high percentage of warmth perception (81.70 ± 11.70% (mean ± SD)).

**Figure 3:**
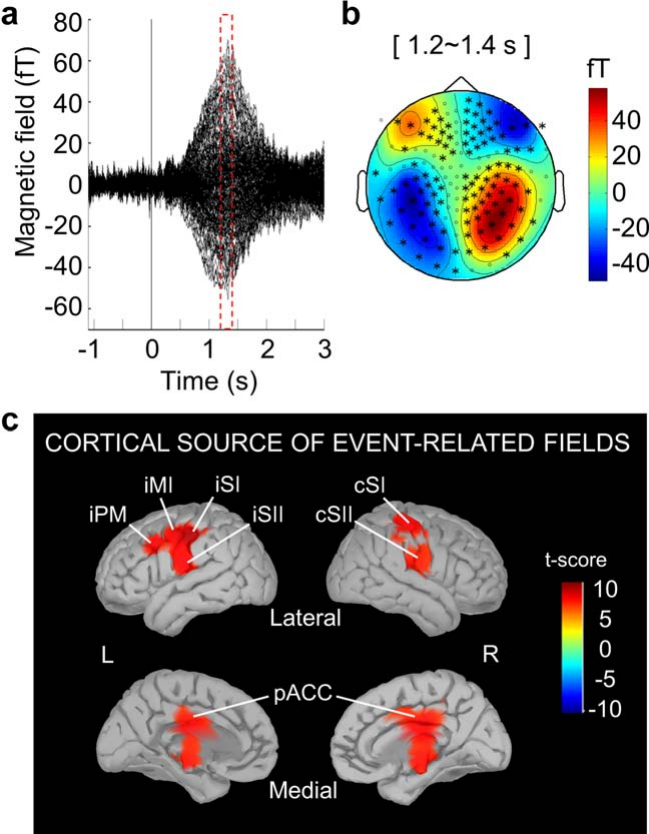
Event‐related fields (ERFs) and their topological and cortical maps (a) Grand‐averaged ERFs evoked by warmth stimulation in 24 participants. (b) Field distribution at the maximum peak of grand averaged ERFs (i.e., at 1.2–1.4 s). The topological map is displayed with axial gradiometer sensors. Black asterisks indicate statistically significant channels (*p* < .05, cluster corrected). (c) Cortical maps at the peak of the warmth‐related ERFs. There was significant activation in the bilateral primary and secondary somatosensory cortex, ipsilateral primary motor cortex, ipsilateral premotor cortex, and posterior part of the anterior cingulate cortex (paired *t* test, *p* < .0001, Bonferroni corrected). cSI, contralateral primary somatosensory cortex; iSI, ipsilateral primary somatosensory cortex; cSII, contralateral secondary somatosensory cortex; iSII, ipsilateral secondary somatosensory cortex; iMI, ipsilateral primary motor cortex; iPM, ipsilateral premotor cortex; pACC, posterior part of the anterior cingulate cortex

Figure 3a demonstrates the grand averaged warmth‐related ERFs across the 24 participants, and the maximum peak of the grand averaged ERFs was found at ∼1.3 s. The mean latency of the individual maximum peak of ERFs across the entire participants was 1.28 ± 0.24 s (mean ± SD). Individual latencies and amplitudes of the ERFs are indicated in the Supporting Information, Table S1.

At the maximum peak of the ERFs (i.e., at 1.2–1.4 s), the field distributions in the MEG sensor space showed a bilateralized magnetic field pattern on the central area (*p* < .05, cluster‐based permutation test) (Figure 3b). As seen in Figure 3c, the cortical sources at the peak of the ERFs were observed in the bilateral primary and secondary somatosensory cortices (SI and SII), ipsilateral primary motor and premotor cortices (MI and PMC), and bilateral pACC (paired *t* test, *p* < .0001, Bonferroni corrected).

### 3.2 Warmth‐related changes in oscillatory activity

**Figure 4:**
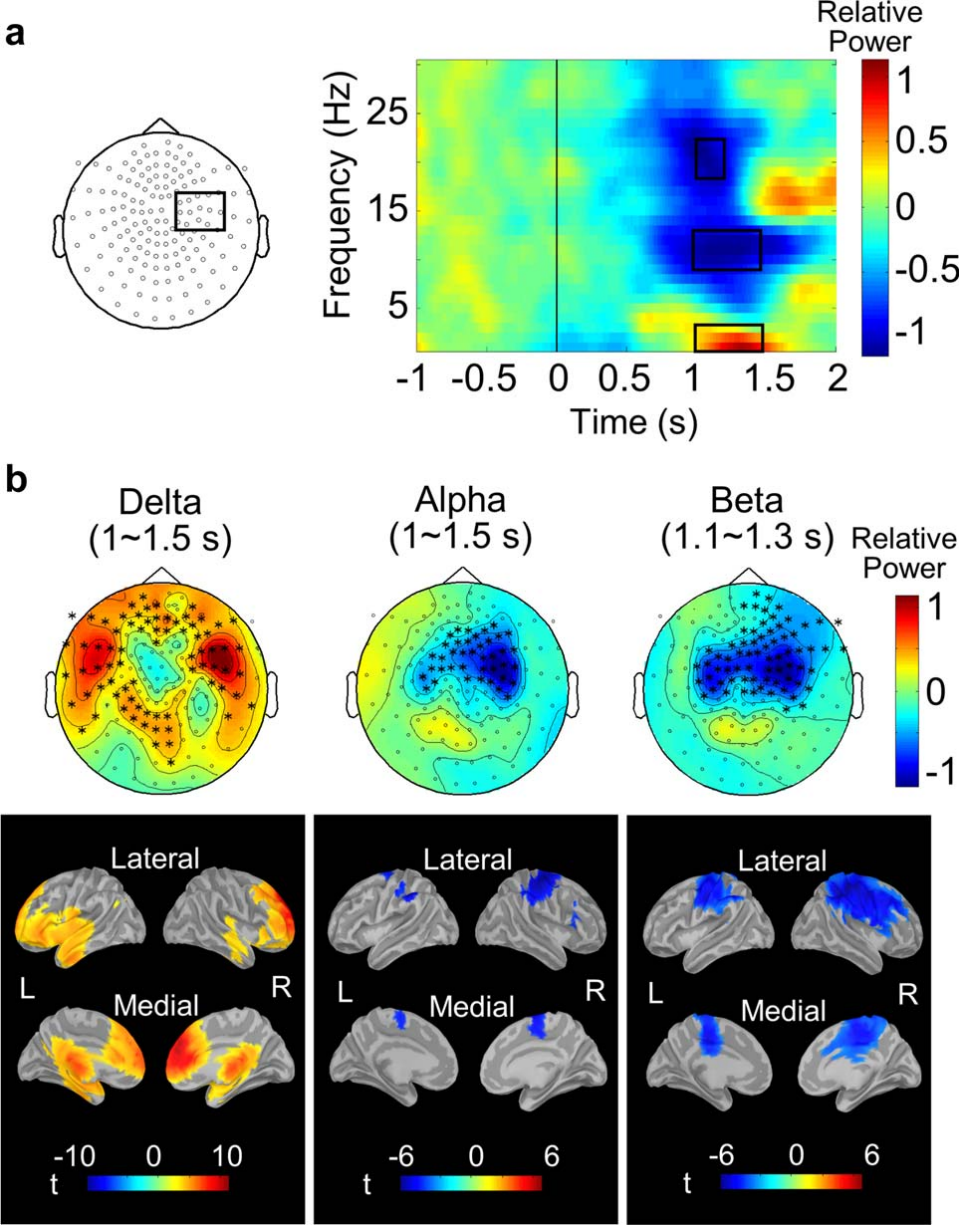
Time‐frequency representations related to the warmth sensation, and topological and cortical mapping of the power changes in delta (1–4 Hz), alpha (8–13 Hz), and beta (18–23 Hz) bands. (a) Grand averaged time–frequency representations of the MEG signals averaged for the planar sensors over the contralateral central area in 24 participants. The activity of the delta band was enhanced, whereas those of the alpha and beta bands were suppressed. The three time‐frequency windows (marked as a black box) were analyzed for topological mapping. (b) Topological maps of the power changes in the delta (1–4 Hz, 1.0–1.5 s), alpha (8–13 Hz, 1.0–1.5 s), and beta (18–23 Hz, 1.1–1.3 s) bands after planar transformation, with the resultant cortical map. Asterisks in the topological maps indicate statistically significant channels. The enhancement of the delta band power was evident in frontal, temporal, and parietal channels (*p* < .05, cluster corrected), and in the frontal, temporal cortices, and cingulate cortices (paired *t* test, *p* < .01, FDR corrected). The oscillatory power suppressions in the alpha and beta bands were observed in the bilateral central channels (*p* < .05, cluster corrected), and in the bilateral sensorimotor cortex and posterior part of the anterior cingulate cortex (paired *t* test, *p* < .01, FDR corrected) [Color figure can be viewed at http://wileyonlinelibrary.com]

To investigate the oscillatory responses to the human cutaneous warmth processing, we calculated the induced oscillatory power changes after warmth stimulation with respect to the pre‐stimulus baseline (i.e., at −1.1 to −0.1 s). Figure 4a demonstrates the time‐frequency representations of the signal from sensors over the contralateral central area. We observed warmth‐induced oscillatory power changes in the delta (1–4 Hz), alpha (8–13 Hz), and beta (18–23 Hz) frequency bands. Figure 4b shows the topological and cortical mapping of the power changes in the delta, alpha, and beta bands. We observed increases in the delta power in the frontal, temporal, and parietal sensors, at 1.0–1.5 s (*p* < .05, cluster corrected). Further, the power of the alpha (1.0–1.5 s) and beta (1.1–1.3 s) bands decreased over the bilateral central sensor area (*p* < .05, cluster corrected).

In the cortical map, increased delta power changes were found in the frontal, temporal cortices, and cingulate cortices (paired *t* test, *p* < .01, FDR corrected). Decreases of the alpha and beta band powers were observed in the bilateral sensorimotor cortices and pACC (paired *t* test, *p* < .01, FDR corrected).

## 4 DISCUSSION

The purpose of this study was to apply MEG to investigate the human neurophysiological response to cutaneous warmth stimulation. A diode laser‐based warmth stimulator enabled us to observe the late‐latency responses of ERFs and oscillatory activities related to the processing of warmth stimuli. We analyzed the topography and cortical mapping of the ERFs and oscillatory band powers.

### 4.1 Latency of event‐related fields and time–frequency representations

Warmth‐related ERFs had a late latency response at ∼1.3 s after stimulation as shown in Figure 3a. In addition, warmth‐induced oscillatory band power changes were prominent at ∼1.0–1.5 s after stimuli onset (Figure 4a). Previous EEG studies reported that ERPs with late latencies, which were called ultra‐late laser evoked potentials, were generated through warmth‐specific C‐fibers (Cruccu et al., 2003; Granovsky et al., 2005; Iannetti et al., 2003; Valeriani et al., 2002; Valeriani et al., 2011) and nociceptive C‐fibers activated by laser stimuli to a tiny skin surface (Kakigi et al., 2003; Opsommer, Weiss, Miltner, & Plaghki, 2001a; Opsommer, Weiss, Plaghki, & Miltner, 2001b; Tran, Lam, Hoshiyama, & Kakigi, 2001; Tran et al., 2002).

Stančák et al. (2007) reported that decreases in the alpha and beta bands power were observed at ∼2 s after the warmth stimulation. The activity of the alpha and beta bands in this study was suppressed 1 s earlier than the latency reported by Stančák et al. (2007). This could be attributed to the characteristics of the warmth stimulator. We used a laser stimulator, which irradiated short duration pulses, whereas the previous study used a contact type heater that provided relatively slow heating.

In the pain study carried out by Raij et al. (2004), a tiny skin surface was stimulated using laser, which activated the nociceptive Aδ‐ and C‐fibers that were responsive to pain. They demonstrated that the laser stimulation of nociceptive Aδ‐ and C‐fibers suppressed alpha and beta band activity. The suppression of the alpha and beta band activity after stimulation of nociceptive C‐fibers in the previous study showed peak latencies, consistent with those observed in our warmth‐related alpha and beta band power suppressions.

The late latencies of the ERFs and oscillatory responses related to the warmth in this study might reflect the characteristics of the C‐fibers, that is, an unmyelinated thin axon (0.2–1.5 μm in diameter) with slow conduction velocity (0.5–2.0 m/s) (Siegel & Sapru, 2006).

### 4.2 Cortical activations related to the warmth

In this study, sources of the ERFs were observed in the bilateral primary and secondary somatosensory cortex (SI and SII), posterior part of the anterior cingulate cortex (pACC), ipsilateral primary motor, and premotor cortex. The previous EEG studies highlighted the activation of the bilateral SII and pACC following warmth (Cruccu et al., 2003; Iannetti et al., 2003; Valeriani et al., 2011) and thermal pain stimulus (Bromm & Chen 1995; Garcia‐Larrea, Frot, & Valeriani, 2003; Valeriani, Restuccia, Barba, Le, & Tonali, 2000). Although the ACC location is a little deep compared to usual MEG sources on cortex, the ACC activation was still detectable by using MEG in the previous pain studies (Inui, Tran, Qiu, Hoshiyama, & Kakigi, 2003; Ploner, Gross, Timmermann, & Schnitzler, 2002). Both warmth and pain inputs are transmitted through two parallel spinothalamic pathways to the SII and pACC (Valeriani et al., 2007, 2011). Valeriani et al. (2002) reported that the EEG signal generated from the ACC was more affected by the attentional distraction from the warmth stimulation, while the EEG signal from the SII was less sensitive to the attentional distraction. They suggested that the SII is related to the sensory‐discriminative function, and the ACC is linked to the affective‐emotional function of warmth sensation.

Both the SI and SII are important brain areas that are involved in cutaneous perception. However, the SI activation to warmth stimuli, which was observed in this study, was not reported in previous EEG studies. fMRI studies, however, have reported the activation of SI in relation to warmth stimulation (Moulton et al., 2005; Peltz et al., 2011). MEG is known to be more sensitive to tangential sources than EEG, and less distorted by different mediums (such as cerebrospinal fluid) in the brain. We suggest that tangential sources in SI might be involved in the processing of warmth sensation.

Meanwhile, previous MEG studies reported that laser irradiation to a tiny skin area stimulated nociceptive C‐fibers, and that SI, SII, and cingulate cortex, similar to cortical sources observed in our warmth study, were activated by nociceptive laser stimuli (Ploner et al., 2002; Qiu et al., 2004). Therefore, we suggest that the neural mechanisms involved in the sensation of warmth might be similar to those involved in the perception of the second pain mediated by nociceptive C‐fibers.

In addition, we observed sources of ERFs in the primary motor and premotor cortices of the left hemisphere. Activations in the primary motor and premotor cortex are lateralized to the left hemisphere, contralateral to the button‐press with the right index finger. In our experimental paradigm, we asked subjects to evaluate warmth perception by pressing the button with right index finger. To avoid the temporal overlap between the warmth perception and execution of the button‐press, an auditory beep sound was added as a cue for the button‐press. Activation in the primary motor and premotor cortex could be affected not by the execution of button‐press but by the motor preparation.

### 4.3 Increases of the delta oscillations related to warmth stimulation

We observed increased delta activity (1–4 Hz) related to the warmth stimulation in the frontal, temporal, and parietal sensors based on planar gradiometer (Figure 4b). Source reconstruction results, at the peak latency (i.e., 1.0–1.5 s), revealed that activity of the delta band was enhanced in the frontal, temporal cortices, and cingulate cortex. Delta activity, however, did not seem to be directly related to the evoked response. The ERFs showed a late latency response characterized by slow oscillations at ∼0.3 Hz, much lower than frequency range used for delta band analysis in this study.

The enhanced delta band activity was also corroborated with previous studies on pain (Hauck, Domnick, Lorenz, Gerloff, & Engel, 2015), and the results from the Sternberg task and Go/No‐Go task (Fernández et al., 2002; Harmony, 2013; Marroquin, Harmony, Rodriguez, & Valdes, 2004). The Sternberg task and Go/No‐Go task are known to activate working memory processes and attentional processes related to internal concentration. Fernández et al. (2002) found the delta power increased during the Sternberg task in the frontal lobes, anterior regions of the temporal lobes, and ACC. During the Go/No‐Go task, delta band activity increased in frontal, temporal, and parietal regions (Harmony, 2013; Marroquin et al., 2004).

Delta band activity was considered to be involved in many cognitive processes (Başar, Başar‐Eroğlu, Karakaş, & Schürmann, 2001). In our experiment, we asked subjects to evaluate the warmth sensation by pressing buttons after the auditory cues. Subjects maintained the perceived presence or absence of warmth sensation in their memory until auditory trigger is delivered. This involves the working memory process, which might be associated with the power increase in the delta band. Harmony (2013) suggested that sustained delta oscillations inhibited the activity of brain networks, which should be inactive during task performance. A functional imaging study revealed that the extended network activated by the perception of pain consists of the somatosensory, insular, cingulate, and prefrontal cortices (Apkarian, Bushnell, Treede, & Zubieta, 2005; Garcia‐Larrea & Peyron, 2013; Tracey & Mantyh, 2007). The enhancement of delta activity in our study could be interpreted as working memory and attentional effect of warmth stimulations.

### 4.4 Decreases of the alpha and beta oscillations related to warmth stimulation

The warmth stimulation suppressed the alpha (8–13 Hz) and beta (18–23 Hz) band activities (Figure 4b). Topographically, the activities on the sensors over the sensorimotor cortex were strongly suppressed. Cortical mapping revealed that the bilateral sensorimotor cortex was the source of alpha and beta suppressions.

These results corroborated several previous studies, which reported the decrease of neuronal oscillations of the alpha and/or beta frequency band in the sensorimotor area during pain sensation (Dowman et al., 2008; Iannetti et al., 2008; Michail et al., 2016; Nir et al., 2012; Ploner et al., 2006; Raij et al., 2004) and touch (Bauer et al., 2006; Michail et al., 2016). For warmth stimulation, the power of alpha oscillations decreased in the contralateral primary somatosensory and primary motor cortex (Stancák, Mlynár, Polácek, & Vrána, 2006; Stančák et al., 2007). Bauer et al. (2006) suggested that mu (8–15 Hz) and beta (15–25 Hz) suppressions are involved in an automatic response from the afferent touch stimulation in the sensorimotor circuit. Hari and Salmelin (1997) highlighted that the sensorimotor alpha oscillations might reflect the functional state of the primary somatosensory cortex. Beta oscillations are related to motor excitation, imagery motor, and action viewing (Hari et al., 1998; Salmelin & Hari, 1994; Schnitzler, Salenius, Salmelin, Jousmäki, & Hari, 1997). Beta band activity might be related to the maintenance of the current sensorimotor or cognitive state (Engel & Fries, 2010) and sensorimotor interaction (Lalo et al., 2007). The suppressions of alpha and beta band powers were hypothesized to be associated with activation or disinhibition of the cortical areas related to the processing of sensory, motor and cognitive operations (Pfurtscheller & Lopes da Silva, 1999). We could assume that the observed decreases of the alpha and beta band powers reflect activation or disinhibition of the bilateral sensorimotor cortex by warmth stimuli.

## 5 CONCLUSIONS

In this study, we evaluated cortical activation of the human brain in relation to warmth sensation using MEG. To conduct this study, we developed a laser warmth stimulator and experimental paradigm.

ERFs and oscillatory power changes in delta, alpha, and beta frequency bands were generated by warmth stimuli. Their late latency response could be attributed to the fact that warmth stimuli are conveyed through warmth‐specific C‐fibers that have a slow conduction velocity. Source analysis of ERFs demonstrated that warmth stimuli evoked activation in the bilateral secondary somatosensory cortex (SII) and posterior part of the anterior cingulate cortex (pACC). Although controversial to previous research, we observed the activation of the bilateral primary somatosensory cortex (SI). Thus, we suggested that the SII, pACC, and SI are involved in the processing of warmth sensations.

Warmth stimuli induced an enhancement in the delta power but reduced power in the alpha and beta bands. The increase of the delta band power lasted from 1.0 to 1.5 s after the onset of warmth stimulation, in the frontal, temporal cortices, and cingulate cortices. These delta band oscillations might be associated with the attentional process and working memory process for warmth sensations. Conversely, the power of the alpha and beta band was decreased at 1.0–1.5 and 1.1–1.3 s, respectively, in the bilateral sensorimotor cortex. These changes might reflect the automatic response to warmth stimulation and promote sensorimotor interaction.

It is generally accepted that ERFs represent an evoked brain activity, while time–frequency representations denote an induced brain activity. Both results showed warmth‐related activation at similar latencies, while the locations of underlying sources were partially different. This implies that the underlying neural mechanisms might be different between the two brain responses.

We hope that brain mapping during warmth processing could improve our neurophysiological understanding of the human cutaneous sense. Further research is required to understand the differences of brain activities between warmth, tactile, and cutaneous pain sensations.

## Supporting information

Additional Supporting Information may be found online in the supporting information tab for this article.

Supporting Information Figure S1

Supporting Information Table S1
