# Supplementary figures and images for: Magnetoencephalographic study of event‐related fields and cortical oscillatory changes during cutaneous warmth processing
Source: Hum Brain Mapp. 2018 Jan 23;39(5):1972–81. doi: 10.1002/hbm.23977 (PMC5947665; doi:10.1002/hbm.23977)

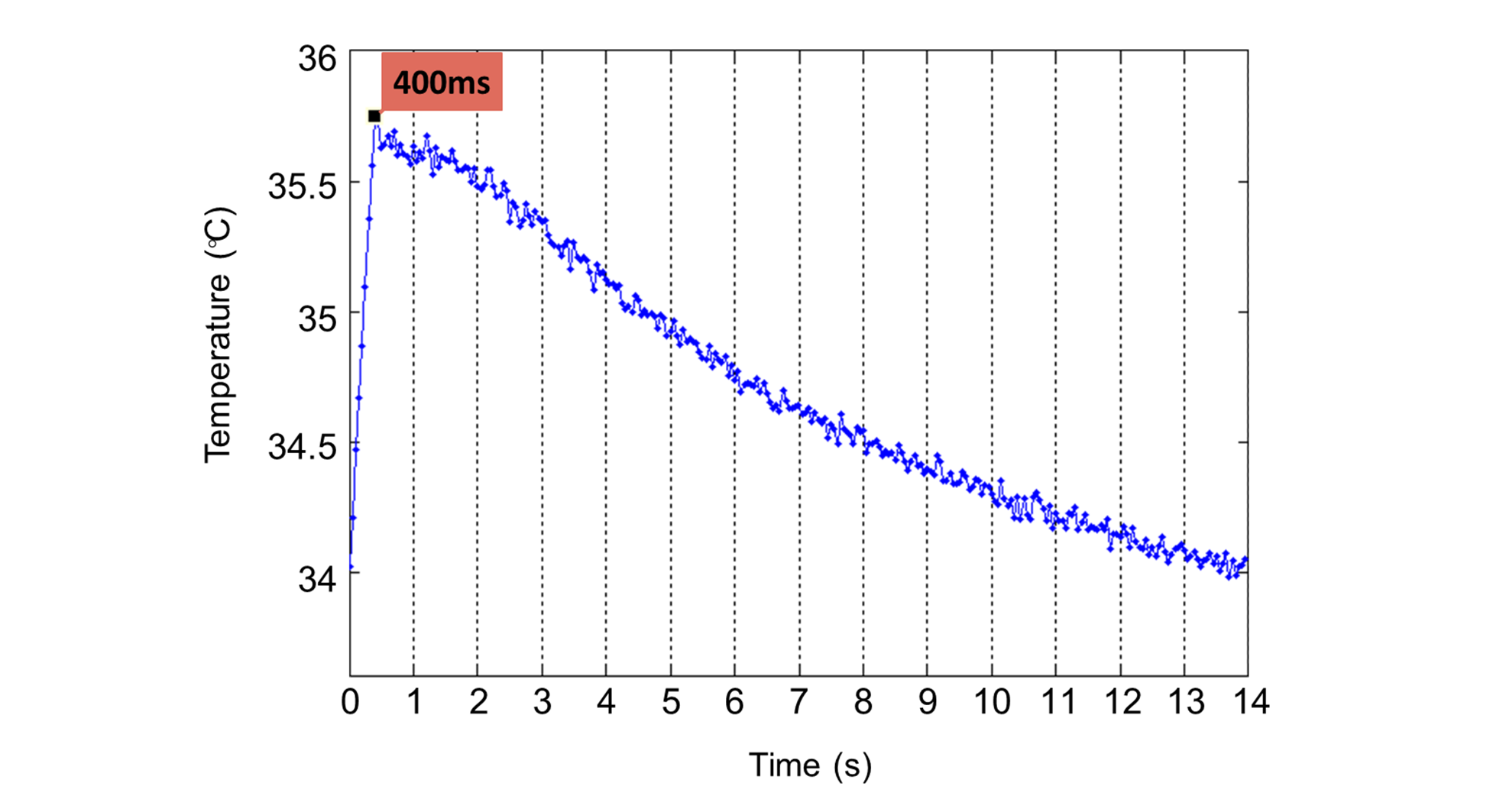

Supplement: Supplementary file 1 — Supporting Information Figure S1 [file HBM-39-1972-s001.TIF]
