# Supplementary material for: Magnetoencephalographic study of event‐related fields and cortical oscillatory changes during cutaneous warmth processing
Source: Hum Brain Mapp. 2018 Jan 23;39(5):1972–81. doi: 10.1002/hbm.23977 (PMC5947665; doi:10.1002/hbm.23977)
Supplement: Supplementary file 2 — Supporting Information Table S1 [file HBM-39-1972-s002.docx]

**TABLE S1. The laser intensity of warmth stimulation, the rate of warmth perception and the individual characteristics of the event-related fields.**

| Participant | Laser Power  (W) | Rate of warmth perception  (%) | Event-related fields | |
| --- | --- | --- | --- | --- |
|  |  |  | Latency (s) | Amplitude (fT) |
| 1 | 3.9 ± 0.3 | 83 ± 6.8 | 1.45 | 153 |
| 2 | 4.4 ± 0.4 | 84 ± 6.7 | 0.90 | 184 |
| 3 | 3.4 ± 0.2 | 98 ± 0.2 | 0.96 | 108 |
| 4 | 4.0 ± 0.2 | 79 ± 3.0 | 1.31 | 167 |
| 5 | 4.5 ± 0.3 | 67 ± 5.6 | 1.42 | 145 |
| 6 | 3.7 ± 0.4 | 82 ± 5.6 | 0.94 | 117 |
| 7 | 4.5 ± 0.4 | 61 ± 5.0 | 1.33 | 164 |
| 8 | 4.7 ± 0.2 | 60 ± 10.9 | 1.61 | 86 |
| 9 | 4.0 ± 0.3 | 82 ± 3.1 | 1.24 | 113 |
| 10 | 4.5 ± 0.4 | 69 ± 6.2 | 1.22 | 130 |
| 11 | 4.9 ± 0.1 | 69 ± 7.2 | 0.90 | 93 |
| 12 | 4.6 ± 0.4 | 82 ± 10.9 | 1.36 | 158 |
| 13 | 3.7 ± 1.0 | 84 ± 9.4 | 1.73 | 69 |
| 14 | 3.0 ± 0.3 | 90 ± 2.6 | 1.49 | 127 |
| 15 | 4.8 ± 0.3 | 74 ± 8.8 | 1.07 | 108 |
| 16 | 4.3 ± 0.7 | 83 ± 13.6 | 1.63 | 216 |
| 17 | 4.0 ± 0.3 | 93 ± 6.6 | 1.48 | 92 |
| 18 | 3.6 ± 0.4 | 93 ± 3.1 | 0.89 | 110 |
| 19 | 4.3 ± 0.5 | 96 ± 2.6 | 1.45 | 110 |
| 20 | 4.9 ± 0.2 | 83 ± 5.1 | 1.38 | 135 |
| 21 | 4.7 ± 0.2 | 83 ± 5.7 | 1.41 | 105 |
| 22 | 3.8 ± 0.2 | 93 ± 1.5 | 1.21 | 113 |
| 23 | 4.1 ± 0.2 | 90 ± 3.5 | 1.33 | 198 |
| 24 | 3.8 ± 0.2 | 82 ± 1.8 | 1.10 | 164 |
| Average | 4.2 ± 1.1* | 82 ± 11.7* | 1.28 ± 0.24* | 132.9 ± 37.1* |

The laser power indicates the intensity of laser warmth stimulation at the warmth threshold of individual participants. The rate of warmth perception corresponds to the ratio of trials where participants pressed the “yes” button, compared to total trials. The values of laser power and rate of warmth perception are presented as mean ± standard deviation (SD). The latencies and amplitudes of the maximum peak of the averaged event-related fields for all participants are also listed.

* The average value of all participants presented as mean ± SD
